# Life expectancy and years of life lost for adults with diagnosed ADHD in the UK: matched cohort study

**DOI:** 10.1192/bjp.2024.199

**Published:** 2025-05

**Authors:** Elizabeth O'Nions, Céline El Baou, Amber John, Dan Lewer, Will Mandy, Douglas G.J. McKechnie, Irene Petersen, Josh Stott

**Affiliations:** UCL Research Department of Clinical, Educational and Health Psychology, Division of Psychology and Language Sciences, London, UK; Bradford Institute for Health Research, Bradford Teaching Hospitals NHS Foundation Trust, Bradford, UK; Institute of Epidemiology and Healthcare, University College London, London, UK; UCL Research Department of Primary Care and Population Health, UCL Medical School, London, UK

**Keywords:** attention deficit hyperactivity disorders, mortality and morbidity, neurodevelopmental disorders, life expectancy, primary care

## Abstract

**Background:**

Nearly 3% of adults have attention-deficit and hyperactivity disorder (ADHD), although in the UK, most are undiagnosed. Adults with ADHD on average experience poorer educational and employment outcomes, worse physical and mental health and are more likely to die prematurely. No studies have yet used mortality data to examine the life expectancy deficit experienced by adults with diagnosed ADHD in the UK or worldwide.

**Aims:**

This study used the life-table method to calculate the life-expectancy deficit for people with diagnosed ADHD using data from UK primary care.

**Method:**

A matched cohort study using prospectively collected primary care data (792 general practices, 9 561 450 people contributing eligible person-time from 2000–2019). We identified 30 039 people aged 18+ with diagnosed ADHD, plus a comparison group of 300 390 participants matched (1:10) by age, sex and primary care practice. We used Poisson regression to estimate age-specific mortality rates, and life tables to estimate life expectancy for people aged 18+ with diagnosed ADHD.

**Results:**

Around 0.32% of adults in the cohort had an ADHD diagnosis, ~1 in 9 of all adults with ADHD. Diagnoses of common physical and mental health conditions were more common in adults with diagnosed ADHD than the comparison group. The apparent reduction in life expectancy for adults with diagnosed ADHD relative to the general population was 6.78 years (95% CI: 4.50 to 9.11) for males, and 8.64 years (95% CI: 6.55 to 10.91) for females.

**Conclusions:**

Adults with diagnosed ADHD are living shorter lives than they should. We believe that this is likely caused by modifiable risk factors and unmet support and treatment needs in terms of both ADHD and co-occurring mental and physical health conditions. This study included data from adults with diagnosed ADHD; the results may not generalise to the entire population of adults with ADHD, the vast majority of whom are undiagnosed.

Attention-deficit and hyperactivity disorder (ADHD) is a neurodevelopmental condition diagnosed based on ‘a persistent pattern (at least 6 months) of inattention and/or hyperactivity-impulsivity that has a direct negative impact on academic, occupational or social functioning’.^1^ It was first included in the DSM in 1968 as ‘hyperkinetic reaction of childhood’.^2^ ADHD persists into adulthood in as many as 90% of those diagnosed as children.^3,4^ The estimated global prevalence of adult ADHD based on 20 representative world mental health surveys is 2.8%.^5^ A UK population-based survey showed that most UK adults self-reporting ADHD characteristics are undiagnosed and not receiving support or intervention,^6^ despite the availability of treatment options that positively impact life outcomes.^7–9^ People with ADHD are disproportionately likely to experience inequality and adversity, including educational under-attainment, unemployment, financial problems, discrimination, contact with the criminal justice system and homelessness.^10–14^ They are also more likely to experience reduced sleep and greater alcohol consumption, and to use substances and smoke.^4,15,16^ On average, people with diagnosed ADHD experience more physical and mental health problems,^17–19^ including cardiovascular disease and associated risk factors.^17,20,21^ They are also at a higher risk of suicide than people not diagnosed with ADHD.^22,23^ A meta-analysis of eight studies (396 488 participants) found that people with diagnosed ADHD are twice as likely than the general population to die prematurely (risk ratio: 2.13; 95% CI 1.13–4.02).^24^ More recent studies report similar results (e.g.^25,26^), although data from Quebec, where the diagnosed ADHD prevalence in young people reached 12.6% by 2017/2018, suggested a lower difference in mortality versus comparison young adults.^27^ Data from the 1958 British birth cohort indicate that being in the top 2% for ‘overreaction’ in childhood (i.e. teacher-report of hostility, restlessness and anxiety about acceptance) was associated with 1.6 times the likelihood of mortality by aged 46, relative to those with a mean level of these traits.^28^ To our knowledge, no study has yet used mortality data to estimate life expectancy in adults diagnosed with ADHD using the life table method. Estimates of years-of-life lost are important because they highlight the impact of unmet needs and health inequalities, and are interpretable to a general audience, as evidenced by their widespread use in advocacy efforts and the mainstream press (e.g.^29^). The aim of this study was to estimate the average years-of-life-lost associated with diagnosed ADHD, based on all-cause mortality data among UK adults from 2000 to 2019.

## Method

### Study design

A matched retrospective cohort study.

### Setting

This study used UK electronic primary care health records from IQVIA Medical Research Data (IMRD). IMRD incorporates data from The Health Improvement Network (THIN), a Cegedim Database. Reference made to THIN is intended to be descriptive of the data asset licensed by IQVIA. IMRD contains anonymised electronic health records drawn from 794 UK primary care practices (c. 10% of all practices), and is largely representative of the UK population.^30^

In the UK, almost all of the population are registered with a National Health Service (NHS) primary care practice, and access is free of charge.^31^ Non-emergency secondary and specialist care is mostly accessed via referral from a primary care practitioner (general practitioner (GP)). Diagnoses (including ADHD) made in secondary care are communicated to the person's GP and entered into their records using a structured system of clinical codes called ‘Read codes’. To meet quality thresholds, all records were required to contain information about age, sex and primary care practice, although there were some missing data on socioeconomic deprivation (see Table 1).

**Table 1:** Participant characteristics: males and females with attention-deficit hyperactivity disorder (ADHD) and their comparison groups

Characteristic	Males		Females	
	Males with ADHD	Comparison males	Females with ADHD	Comparison females
*N* individuals	23 377	233 770	6662	66 620
*N* practices	778	778	749	749
*N* deaths (%)	193 (0.83)	1219 (0.52)	148 (2.22)	902 (1.35)
Median age at death (IQR)	38.93 (24.74–60.94)	42.12 (23.95–74.60)	77.22 (62.68–85.55)	82.97 (70.52–89.69)
Age at cohort entry				
Median age at entry (IQR)	18.95 (18.00–24.54)	18.95 (18.00–24.54)	22.10 (18.00–31.50)	22.10 (18.00–31.50)
18–24 years	17 881 (76.49)	178 810 (76.49)	4095 (61.47)	40 950 (61.47)
25–34 years	3630 (15.53)	36 300 (15.53)	1205 (18.09)	12 050 (18.09)
35–44 years	1020 (4.36)	10 200 (4.36)	581 (8.72)	5810 (8.72)
45–54 years	498 (2.13)	4980 (2.13)	323 (4.85)	3230 (4.85)
55–64 years	175 (0.75)	1750 (0.75)	184 (2.76)	1840 (2.76)
65+ years	173 (0.74)	1730 (0.74)	274 (4.11)	2740 (4.11)
Median length of follow-up (IQR)	2.54 (1.03–5.53)	3.24 (1.34–6.18)	2.17 (0.80–4.62)	2.56 (1.06–5.38)
Socioeconomic status (Townsend score) (%)				
1: least deprived	2884 (12.34)	41 902 (17.92)	832 (12.49)	11 359 (17.05)
2	2895 (12.38)	38 325 (16.39)	830 (12.46)	10 592 (15.90)
3	4121 (17.63)	42 820 (18.32)	1074 (16.12)	11 860 (17.80)
4	4755 (20.34)	41 458 (17.73)	1324 (19.87)	11 596 (17.41)
5: most deprived	3854 (16.49)	28 804 (12.32)	1115 (16.74)	8794 (13.20)
Missing	4868 (20.82)	40 461 (17.31)	1487 (22.32)	12 419 (18.64)
Year of cohort entry				
2000–2009	6811 (29.14)	68 110 (29.14)	1731 (25.98)	17 310 (25.98)
2010–2019	16 566 (70.86)	165 660 (70.86)	4931 (74.02)	49 310 (74.02)

Note: Age at cohort entry has a 6-month margin of error, because only year of birth is available for over-18 s in IQVIA Medical Research Data (IMRD). Ages are identical for matched groups because we matched by year of birth.

### Ethical approval

IMRD holds ethical approval to collect and supply data for research purposes from the National Health Service (NHS) London – South East Research Ethics Committee (reference 18/LO/0441). Use of the IMRD for this study was obtained and approved by IQVIA World Publications Scientific Review Committee in March 2022 (reference 22SRC012).

### Study population

We included people who had a diagnostic code indicative of ADHD (e.g. attention-deficit disorder, attention-deficit with hyperactivity) based on an existing codelist.^32^ The diagnosis could have been recorded in the person's medical record at any time. Historically used terms indicating ADHD such as hyperkinetic syndrome were included.^2^ The codelist is provided as a Supplemental file.

For participants with ADHD, the cohort entry date (i.e. the start of their observation period) was the latest of the following dates: the date of their ADHD diagnosis, the date on which their primary care practice met quality criteria for electronic healthcare record-keeping (acceptable mortality recording^33^ and acceptable computer usage^34^), their date of registration at the practice +6 months, the date that their primary care practice began contributing data to IMRD and 1 January 2000. The participant's date of cohort exit (i.e. the end of their observation period) was the earliest of the following: their date of death (if applicable), the date of their deregistration from the practice, the date that their practice no longer contributed data to IMRD and 16 January 2019. This approach assumes that someone who is diagnosed with ADHD has ADHD for the rest of their life, but does not include time prior to ADHD diagnosis to avoid inclusion of immortal time in the analysis.

For each individual diagnosed with ADHD, we sampled ten comparison people matched by age, sex and primary care practice. We used exposure density sampling to identify these individuals and determine their cohort entry dates to minimise the impact of time-dependent bias.^35^ For each individual joining the cohort who had been diagnosed with ADHD, we randomly sampled comparison individuals who did not have an ADHD diagnosis on the date of cohort entry for the corresponding participant with ADHD. We then assigned the comparison participant the same cohort entry date as their corresponding participant with ADHD. Cohort exit dates were identified in the same way as for participants with ADHD. Supplementary eFigs 1 and 2 available at https://doi.org/10.1192/bjp.2024.199 describe the identification of eligible participants and cohort entry dates.

### Study variables

The outcome was all-cause death. In the UK, registration of a death triggers an update in the NHS Personal Demographic Service, which is then automatically updated in the practice's electronic records. Prior to this system, GPs were notified about patient deaths by the local Health Authority, secondary care bereavement offices and/or by family members. Comparisons between primary care and ONS data have shown that 98.2% of recorded deaths can be identified from primary care records.^36^ Further information about identification of death records is provided in the Supplementary Methods and eFig. 3.

Previous studies investigating mortality in ADHD have reported an increased likelihood of deaths from unnatural causes, including suicide or accidents.^24^ As a sensitivity analysis, we performed an additional search using a codelist to identify potentially fatal incidents, in case such deaths were not captured by the process described above. Further information is provided in the Supplementary Methods. We report results for analyses including these possible deaths in Supplementary eTables 3–6.

We also extracted data on: socioeconomic deprivation (Townsend quintile); diabetes; hypercholesterolaemia; ischaemic heart disease; hypertension; chronic respiratory disease; epilepsy; anxiety; depression; severe mental illness; self-harm/suicide; autism, intellectual disability; personality disorder; current smoking and potentially harmful alcohol use. For health conditions, we report the difference in rates of diagnosis of these conditions in the ADHD and comparison groups at the start of follow-up (Table 2). For deceased individuals, we report the proportion who had ever had a code indicating these conditions in Supplementary eTables 1–4.

**Table 2:** Pre-existing diagnosed health conditions/lifestyle variables in attention-deficit hyperactivity disorder (ADHD) versus comparison groups at baseline

Variable	Males			Females		
	Males with ADHD *n* (%)	Comparison males *n* (%)	Baseline odds ratio for ADHD versus comparison group (95% CI)	Females with ADHD *n* (%)	Comparison females *n* (%)	Baseline odds ratio for ADHD versus comparison group (95% CI)
Physical health condition						
Diabetes	359 (1.54)	3067 (1.31)	1.17 (1.05–1.31)	248 (3.72)	1655 (2.48)	1.52 (1.32–1.74)
Hypercholesterolemia	221 (0.95)	1904 (0.81)	1.16 (1.01–1.34)	173 (2.60)	1368 (2.05)	1.27 (1.08–1.5)
Hypertension	353 (1.51)	2794 (1.20)	1.27 (1.13–1.42)	306 (4.59)	2433 (3.65)	1.27 (1.12–1.44)
Ischaemic heart disease	88 (0.38)	677 (0.29)	1.30 (1.03–1.63)	74 (1.11)	489 (0.73)	1.52 (1.17 - 1.95)
Chronic respiratory disease	583 (2.49)	4456 (1.91)	1.32 (1.2–1.44)	204 (3.06)	1488 (2.23)	1.38 (1.19–1.61)
Epilepsy	789 (3.38)	2662 (1.14)	3.03 (2.8–3.29)	255 (3.83)	751 (1.13)	3.49 (3.01–4.04)
Mental health/neurodevelopmental condition						
Anxiety	2628 (11.24)	10 198 (4.36)	2.78 (2.65–2.91)	1461 (21.93)	7026 (10.55)	2.38 (2.24–2.54)
Depression	3403 (14.56)	12 237 (5.23)	3.08 (2.96–3.21)	2080 (31.22)	9822 (14.74)	2.63 (2.48–2.78)
SMI	707 (3.02)	1083 (0.46)	6.70 (6.08–7.38)	307 (4.61)	383 (0.57)	8.35 (7.15–9.76)
Self-harm/suicide	2433 (10.41)	4593 (1.96)	5.80 (5.51–6.10)	1265 (18.99)	3086 (4.63)	4.83 (4.49–5.18)
Autism	2401 (10.27)	2937 (1.26)	9.00 (8.51–9.52)	447 (6.71)	217 (0.33)	22.01 (18.61–26.03)
Intellectual disability	1419 (6.07)	2240 (0.96)	6.68 (6.24–7.15)	404 (6.06)	391 (0.59)	10.93 (9.47–12.63)
Personality disorder	486 (2.08)	407 (0.17)	12.17 (10.64–13.93)	302 (4.53)	252 (0.38)	12.51 (10.52–14.86)
Lifestyle variables						
Smoking	9397 (40.20)	46 950 (20.08)	2.67 (2.60–2.75)	2812 (42.21)	16 878 (25.33)	2.15 (2.04–2.27)
Potentially harmful alcohol use	1717 (7.34)	8291 (3.55)	2.16 (2.04–2.28)	456 (6.84)	2665 (4.00)	1.76 (1.59–1.96)

Note: SMI = severe mental illness.

Socioeconomic deprivation was measured using Townsend scores, derived for each area using 2001 census data.^37^ Each output area corresponds to approximately 150 households. For each area, statistics were calculated based on the percentage of households without access to a car, that were not owner-occupied, and household overcrowding, as well as the percentage of the economically active population aged 16–74 who were unemployed. For each variable, the scores were converted from exact scores into quintiles consisting of five groups of equal size to indicate local deprivation level. UK postcodes were then matched to output area Townsend deprivation quintile. A Townsend score of 1 indicates the least deprived quintile, and a score of 5 the most.

### Statistical analysis

We first calculated the number of deaths and person-years of observation, stratified by presence of an ADHD diagnosis, sex and single-year-of-age from 18 to 100, accounting for time-varying age^1^.^38^ We then calculated the mortality rates in each stratum. We used a Poisson model to estimate the mortality rate by single year-of-age in each group (men with ADHD, women with ADHD, comparison men, comparison women). The purpose of using a model rather than the observed rates is that we expected mortality rates to change smoothly with age, and this assumption adds power to the analysis. The dependent variable was the count of deaths, and the independent variables were age (linear and quadratic terms) and an offset for the log observation time.

We used these modelled rates to estimate life expectancy at age 18 years using the period life table method described by the Office for National Statistics.^39^ We estimated 95% CI by simulating a distribution of mortality rates using the uncertainty in the Poisson model, and then estimating life expectancy for each simulation. Data preparation was performed using Stata 16, and data analyses using R version 4.2.2. For ease of interpretation we report the total expected life expectancy at age 18 (i.e. we added 18 to the life expectancy at age 18).

### Patient and public involvement

People with lived experience of ADHD, plus their family members, were involved in the design and conduct of this research. They gave positive feedback on the appropriateness and usefulness of the research question addressed here, and their feedback informed the preparation of the manuscript.

## Transparency declaration

The lead author affirms that the manuscript is an honest, accurate and transparent account of the study being reported, and that no important aspects of the study have been omitted.

## Results

### Participants

Of the 9 561 450 people contributing eligible person-time, we identified 30 529 people with an ADHD diagnosis in their electronic health records (0.32% of the sample). Therefore, across the time-period studied, around 1 in 300 people had an ADHD diagnosis, approximately 1 in 9 of the likely true number of people with ADHD based on population-based surveys (2.8%).^5^

A total of 162 people with ADHD were excluded because they had no date associated with their diagnosis, and for 328 there were too few matched comparison participants available within their primary care practice (Supplementary eFigure 1). Data from 30 039 people with diagnosed ADHD (23 377 males and 6662 females) were included, plus 300 390 matched comparison people (233 770 males and 66 620 females). Sample characteristics are provided in Table 1.

For participants with ADHD, the median year when their diagnosis was made was 2003 (inter-quartile range (IQR): 1999–2009); 2003 for males (IQR: 1998–2008) and 2006 for females (IQR: 2000–2012). The median age at cohort entry was 18.95 years (IQR: 18.00–24.54) for males and 22.10 years (IQR: 18.00–31.50) for females. In total, 76.49% of males and 61.47% of females entered the study cohort between the ages of 18 and 24 years. The proportion entering the study after 2010 was 70.86% for males, and 74.02% for females.

All co-occurring conditions that we examined at baseline were more common among participants with ADHD than comparison participants (Table 2).

### Mortality rates and ratios

**Fig. 1:**
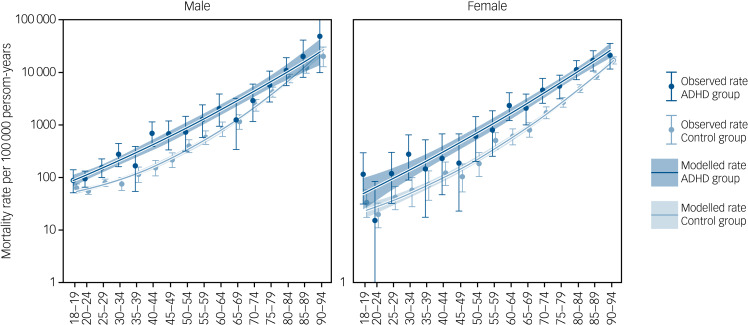
Mortality rate per 100 000 person-years for participants with attention-deficit hyperactivity disorder (ADHD) versus comparison participants. Error bars and shaded areas indicate 95% CI.

Among male participants with diagnosed ADHD, 193 of 23 377 (0.83%) died; relative to 1219 of 233 770 (0.52%) of comparison males (see Table 1). Among female participants with diagnosed ADHD, 148 of 6662 (2.22%) died, relative to 902 of 66 620 (1.35%) of comparison females (Supplementary eTable 2).^2^ Mortality rates increased exponentially with age (Fig. 1). The apparent estimates indicate that males with diagnosed ADHD were 1.89 (95% CI: 1.62–2.19) times as likely to die during follow-up versus comparison males, and females diagnosed with ADHD were 2.13 (1.79–2.53) times as likely. Mortality rates by age group are presented in Table 3.

**Table 3:** Mortality rates by age group and sex (with 95% CIs)

Age band	Mortality rate per 100 000	
	Males with diagnosed ADHD	Comparison males
18–24	91.71 (68.07–120.91)	57.37 (51.41–63.83)
25–34	185.60 (134.32–250.00)	78.28 (68.27–89.33)
35–44	385.73 (235.61–595.73)	130.03 (102.27–162.99)
45–54	689.43 (415.08–1076.62)	286.28 (230.49–351.49)
55–64	1545.42 (915.91–2442.43)	673.74 (540.36–830.07)
65+	4793.19 (3468.86–6456.40)	3805.61 (3445.26–4193.42)

	Females with diagnosed ADHD	Comparison females
18–24	49.68 (16.13–115.95)	24.12 (15.89–35.09)
25–34	172.54 (78.89–327.53)	46.07 (30.62–66.59)
35–44	184.58 (59.93–430.74)	96.47 (64.10–139.42)
45–54	370.33 (148.89–763.03)	136.59 (90.76–197.40)
55–64	1477.43 (860.66–2365.51)	544.21 (422.60–689.91)
65+	6834.67 (5590.08–8273.78)	3975.05 (3690.54–4275.67)

### Life expectancy

**Fig. 2:**
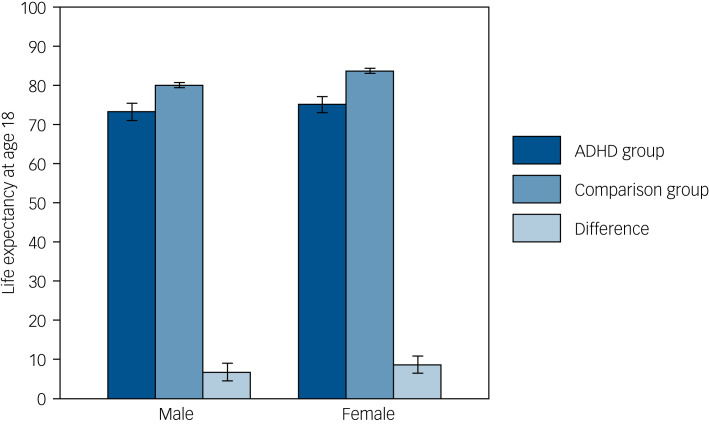
Life expectancy for participants with attention-deficit hyperactivity disorder (ADHD) stratified by sex, compared with matched comparison participants without a diagnosis of ADHD. Error bars indicate 95% CI.

The apparent total life expectancy estimate for male participants with diagnosed ADHD was 73.26 years (95% CI: 71.06–75.41), compared with 80.03 years (95% CI: 79.34–80.74) for comparison males. For female participants with diagnosed ADHD, the apparent total life expectancy estimate was 75.15 years (72.99–77.11), compared with 83.79 years (95% CI: 83.12–84.44) for comparison females. Thus, our apparent estimates suggest that having a diagnosis of ADHD was associated with 6.78 (95% CI: 4.50–9.11) years-of-life lost in males and 8.64 (95% CI: 6.55–10.91) years-of-life lost in females. Life expectancies are presented in Fig. 2.

### Sensitivity analysis

Including both definite and possible deaths had negligible impact on both the mortality ratios and the life expectancies and years-of-life lost experienced by people diagnosed with ADHD (see Supplementary eTables 5 and 6).

## Discussion

The aim of this study was to provide the first estimate of life expectancy for UK adults diagnosed with ADHD. We found that adults diagnosed with ADHD are living shorter lives than they should: the apparent years of life lost for males was around 7 years, and for females, 9 years, compared with the general population. We believe that this is unlikely to be because of ADHD itself and likely caused by modifiable factors such as smoking, and unmet mental and physical health support and unmet treatment needs. The findings illustrate an important inequity that demands urgent attention.

### Comparison with other studies

The mortality ratio derived from a recent meta-analysis including data from the USA, Denmark, Norway, Finland, Sweden and Taiwan is similar to that reported here.^24^ This suggests that premature mortality experienced by people with diagnosed ADHD in the UK is similar to other high-income countries.^10^ Data from the 1958 birth cohort study suggest a more modest increase in mortality than we observed: by age 46, the predicted probability of death adjusted for gender was 2.1% for those with the population-average level of ‘overreaction’ (which included ADHD characteristics), compared with 3.4% for those scoring 2 standard deviations above the mean (the 98th percentile);^28^ this is equivalent to a 1.6-fold increased risk of premature death.

Only one other study has thus far estimated life expectancy for people with an ADHD diagnosis. Critically, this study did not use mortality data, but instead used self-reported education, occupation, health and lifestyle variables at age 27 to predict life expectancy using life tables.^15^ There was an estimated 8-year reduction in life expectancy associated with childhood hyperactivity based on these data. For the subset of previously diagnosed hyperactive children who met full ADHD criteria as adults, the estimated reduction was 13 years. Given the more stringent diagnostic criteria for ADHD at the time of recruitment to this study, participants may overrepresent those with higher support needs, relative to people meeting current ADHD criteria on average.

### Implications

The evidence that people with diagnosed ADHD are living shorter lives than they should is extremely concerning, and highlights unmet support needs that require urgent attention. People with ADHD are more likely to experience various forms of adversity.^10–14^ Self-management of ADHD / associated mental health challenges with substance use, smoking, excess risk taking or compulsive behaviour^10,40–43^ may increase the likelihood of premature death. These factors are likely to affect many causes of morbidity and mortality. A recent Swedish register-based study with >5 million adults reported that diagnosed ADHD was associated with a two-fold increase in the rate of cardiovascular disease compared with the general population.^21^

At present, there is a dearth of specialist services to support adults with ADHD in the UK.^10^ A population-based study revealed that those with ADHD characteristics were more likely than comparison adults to have accessed support, or to have requested a particular treatment that they did not subsequently receive.^6^ This suggests that they are presenting to services, but services are not equipped to offer support.^10^ Recent work investigating the views of UK primary care practitioners identified a lack of effective systems and pathways for people with ADHD, leaving practitioners feeling ill-equipped to meet their needs.^44^

Recent policy initiatives motivated by the reduced life expectancy experienced by autistic people are emerging,^45,46^ yet no comparable initiatives have begun for ADHD. The present findings suggest that there is an urgent need for initiatives to address unmet support needs. As well as ADHD-specific support, this may include approaches to improve awareness of physical and mental health conditions that are more common in people with ADHD (Table 2), and promotion of timely access to mental health support and smoking cessation services.

### Strengths and limitations

A strength of this study is that both the diagnosed ADHD and matched comparison participants were registered at the same general practices, meaning that regional variation in mortality rates, or practice-level variability in mortality recording, would have affected both groups equally. Limitations include the lack of information about cause of death, meaning that it was not possible to attribute years of life lost to different causes. Our cohort was derived from a primary care database that only includes the date of death and does not include other information from death certificates such as the cause of death. Although we did have information about comorbidities from primary care data, most diseases were rare because of the young average age of participants, and prevalent diseases may be unrelated to the cause of death. Many of the deaths in younger age groups are likely because of suicides, accidents, drugs and alcohol, but this would be difficult to determine from diagnosed comorbidities. A further limitation is the wide confidence intervals around the point-estimates. This was because of the relatively small proportion of people with an ADHD diagnosis, and the fact that most are young, resulting in comparatively few deaths during the observation period.

The dearth of specialist services for adult ADHD assessment in the UK^10,47^ means that diagnosed adults may overrepresent those who have co-occurring mental health and/or neurodevelopmental conditions, which brought them into contact with specialists who diagnosed their ADHD. This would lead to differential exposure misclassification: the association between these conditions and reduced life expectancy may confound the results and lead to an overestimation of years-of-life-lost. As such, there is a need for further estimates of life expectancy to be made using population-based surveys including diagnosed and undiagnosed individuals (e.g. Jokela et al^28^).

While population-based studies can offer insights into both mortality and mediating factors such as social health determinants, they also have limitations that impact generalisability. ADHD characteristics and co-occurring conditions may differentially impact participation/loss to follow-up. There is often a lack of information about behaviours in different contexts that are needed to inform clinical diagnosis. Population-based studies often exclude people with co-occurring intellectual disabilities and/or additional support needs, which are associated with ADHD.^48^ Therefore, a triangulation of evidence across diagnosed samples and population-based surveys is needed to provide a more complete picture of premature mortality in people with ADHD.

In the present study, we did not adjust for socioeconomic status (SES), as we believe that SES is best understood as part of the causal pathway between ADHD and premature mortality (i.e. SES is a mediator). A recent UK population-based longitudinal study found that c. 20% of young people not accessing education or training, who were receiving state financial support and who were homeless had ADHD,^49^ suggesting that ADHD has important effects on SES and social exclusion. Evidence from Swedish registries also indicates that adults with ADHD are more likely to experience unemployment and accumulating financial distress,^50^ increasing their likelihood of suicidality.^51^ We believe that supporting adults with ADHD to access employment and preventing discriminatory practices^11^ are potential mechanisms by which the life expectancy deficit could be reduced.

Race/ethnicity is a potential confounder, which was not included in our models because of the non-random missingness of ethnicity information captured in primary care data.^52^ USA data indicate ethnic disparities in diagnosis and treatment of ADHD,^53^ though at present, no studies have examined this in the UK. Research using advanced methods to identify missing ethnicities (e.g. Pham et al^52^) is needed to address questions around ADHD, ethnicity and premature mortality using data from primary care. We were also unable to examine mortality in gender diverse people with ADHD, as gender diversity was not coded in the database.

A further limitation is that the present findings may not generalise to other countries, time periods or settings. There is a potential for new initiatives, such as improved service pathways and awareness of health needs among people with ADHD to reduce the risk of premature mortality. Alternatively, widening societal inequalities may exacerbate challenges for people with ADHD who are disproportionately likely to experience adversity.

## Supporting information

O'Nions et al. supplementary material 1 O'Nions et al. supplementary material

O'Nions et al. supplementary material 2 O'Nions et al. supplementary material

O'Nions et al. supplementary material 3 O'Nions et al. supplementary material

O'Nions et al. supplementary material 4 O'Nions et al. supplementary material

O'Nions et al. supplementary material 5 O'Nions et al. supplementary material

## Data Availability

The data are not publicly available because of restrictions.

## Footnotes

1 This means that an individual who joins on their 18th birthday and is followed for 3 years contributes 1 year at age 18, 1 year at age 19 and 1 year at age 20.

2 The higher proportion of females with ADHD and comparison females who died during follow-up reflects the different age distribution of females diagnosed with ADHD, and thus the matched comparison females, compared with the males.
