# Supplementary material for: Life expectancy and years of life lost for adults with diagnosed ADHD in the UK: matched cohort study
Source: Br J Psychiatry. 2025 Jan 23;226(5):261–8. doi: 10.1192/bjp.2024.199 (PMC7617439; doi:10.1192/bjp.2024.199)
Supplement: O'Nions et al. supplementary material 1 — O'Nions et al. supplementary material [file S0007125024001995sup001.docx]

**Supplementary Figures for “Life expectancy and years of life lost for adults with diagnosed ADHD in the UK: a matched cohort study”**

Contents

[eFigure 1: Flow diagram for practice/ participant exclusions: main analysis 2](#_Toc146188931)

[eFigure 2: Simplified schematic description of steps to apply EDS to identify the matched groups & start dates 3](#_Toc146188932)

[eFigure 3: Identification of deaths 5](#_Toc146188933)

## eFigure 1: Flow diagram for practice/ participant exclusions: main analysis

| 794 practices |  | 2 excluded: Missing Acceptable Computer Usage/ Acceptable Mortality Recording date |
| --- | --- | --- |
|  |  |  |
| 792 practices  18,241,856 people |  | 2,517,068 excluded due to poor record quality or not being permanently registered. |
|  |  |  |
| 15,724,788 people |  | 6,163,338 excluded due to no data after the point that the practice met data quality thresholds for electronic recording of patient data and/or no person-time after 18^th^ birthday and/or no person-time after 01/01/2000 |
|  |  |  |
| 9,561,450 people |  | 162 excluded due to having a record of ADHD with no date |
|  |  |  |
| 30,367 people with an ADHD diagnosis prior to, or during follow-up |  | 9,530,905 people with no ADHD record at any time; 16 people with a record of ADHD after the end of follow-up. |
|  |  |  |
| **People with a record of ADHD**  30,367 people with an ADHD diagnosis prior to, or during follow-up |  | **Sampling pool for identification of matched participants**  9,530,905 people with no ADHD record at any time.  3,787 people contribute person-time prior to an ADHD diagnosis. |
|  |  |  |
| 30,367 to be matched  328 had insufficient matches |  |  |
|  |  |  |
| **30,039 matched 10:1** |  | **300,390 matches** |

## eFigure 2: Simplified schematic description of steps to apply EDS to identify the matched groups & start dates

The schematics below depict a simplified version of Exposure Density Sampling (EDS). Rows represent different individuals within a primary care practice, registered and contributing data from 2001 up until 2011 or the point at which they left the practice.

**Step 1:** We allocated person-time (after patient registration and once data quality thresholds have been met) to one of 3 categories, designated by the different colours:

|  | **2001** | **2002** | **2003** | **2004** | **2005** | **2006** | **2007** | **2008** | **2009** | **2010** |  |  | **Key** |  |  |
| --- | --- | --- | --- | --- | --- | --- | --- | --- | --- | --- | --- | --- | --- | --- | --- |
| **A** | **.** |  |  |  |  | **X** |  |  |  |  |  | **.** | **Date of registration** | | |
| **B** | **.** | **X** |  |  |  |  |  |  |  |  |  | **X** | **ADHD diagnosis** | | |
| **C** | **.** |  |  |  |  |  |  | **X** |  |  |  |  |  |  |  |
| **D** | **.** |  |  |  |  |  |  |  |  |  |  |  | **ADHD person-time** | | |
| **E** | **.** |  |  |  |  |  |  | **X** |  |  |  |  | ***Potentially* eligible person-time** | | |
| **F** | **.** |  |  |  |  |  |  |  |  |  |  |  | ***Ineligible person-time*** | | |
| **G** | **.** |  |  |  |  |  |  | **X** |  |  |  |  |  | | |
| **H** | **.** |  |  |  |  |  |  |  |  |  |  |  |  | | |

**Step 2:** For each person with an ADHD diagnosis, we identified a set of matched people who were in the database & did not have a record of ADHD on the date of the person with ADHD’s diagnosis. We gave them the same start (“index”) date as their ADHD counterpart (the date of the person with ADHD’s diagnosis; or their entry to the database if they already had a diagnosis when they registered at a contributing practice).

|  | **2001** | **2002** | **2003** | **2004** | **2005** | **2006** | **2007** | **2008** | **2009** | **2010** |  |  |  |  |  |
| --- | --- | --- | --- | --- | --- | --- | --- | --- | --- | --- | --- | --- | --- | --- | --- |
| **A** | **.** |  |  |  |  | **X** |  |  |  |  |  |  |  |  |  |
| **P** | **.** |  |  |  |  |  |  |  |  |  |  |  |  |  |  |
| **Z** | **.** |  |  |  |  |  |  |  |  |  |  |  |  |  |  |
| **B** | **.** | **X** |  |  |  |  |  |  |  |  |  |  |  |  |  |
| **A** | **.** |  |  |  |  | **X** |  |  |  |  | *Note that A, who is later diagnosed with ADHD, is sampled as a match for B, and is censored from the comparison group prior to receiving their ADHD diagnosis.* | | | | |
| **H** | **.** |  |  |  |  |  |  |  |  |  |  |  |  |  |  |
| **C** | **.** |  |  |  |  |  |  | **X** |  |  |  |  |  |  |  |
| **X** | **.** |  |  |  |  |  |  |  |  |  |  |  |  |  |  |
| **P** | **.** |  |  |  |  |  |  |  |  |  |  |  |  |  |  |

This method allowed us to identify matched participants and start-dates for our cohort to calculate person-years at risk without introducing immortal time bias.

## eFigure 3: Identification of deaths

| 330,429 individuals |  | 2,403 deaths identified based on information from IMRD |
| --- | --- | --- |
|  |  |  |
| Additional search of medical records for codes indicating death, e.g. “cause of death”, “patient died”, “post mortem exam” |  | 73 further deaths identified that were not identified by IMRD because they had not been flagged as transferred out of the practice due to their death. |
|  |  |  |
| Medical and prescribing records for all deceased individuals checked to ensure no consulting/prescribing > 6 months after the date of death. |  | 14 individuals had records of continued consulting/prescribing after their supposed death, suggesting that their death records were erroneous. These individuals were recoded as not deceased at cohort exit. |
|  |  |  |
|  |  | 2,462 deaths identified |
