# Supplementary material for: Life expectancy and years of life lost for adults with diagnosed ADHD in the UK: matched cohort study
Source: Br J Psychiatry. 2025 Jan 23;226(5):261–8. doi: 10.1192/bjp.2024.199 (PMC7617439; doi:10.1192/bjp.2024.199)
Supplement: O'Nions et al. supplementary material 2 — O'Nions et al. supplementary material [file S0007125024001995sup002.docx]

**Supplemental methods: Life expectancy and years of life lost for adults with diagnosed ADHD in the UK: a matched cohort study**

***Exposure density sampling***^1^

A diagram of the sampling process is provided in eFigure 2. For people with diagnosed ADHD, their index date (i.e. the beginning of follow-up time) was the latest of their date of diagnosis, their date of registration at the practice, and the date that their practice met record-keeping quality criteria. Age, sex, and general practice-matched comparison individuals were sampled from those registered at the practice prior to, or on the date of, the person with diagnosed ADHD’s index date. Matched comparison individuals were assigned the same index date as their matched ADHD counterparts. If a person was sampled as a comparison individual and was then diagnosed with ADHD during follow-up, they were censored from the comparison group the day before their diagnosis.

Follow-up ended on the earliest of each person's recorded date of death, the date that they were transferred out of the practice, or the last date that their practice contributed data to IMRD.

***Acceptable mortality recording***

We included person-time subsequent to each practice reaching the threshold for both Acceptable Computer Usage (ACU)^2^ and Acceptable Mortality Recording (AMR).^3^ The date at which the practice achieves ACU is the date at which the average annual rates of recording included least one medical record, one alternative health data record, and two prescription records per patient.^2^ The date at which the practice achieves AMR is determined based on a review of trends in death reporting for each practice against the predicted number of deaths given the practice’s demographics based on national statistics.^3^ IMRD provides a date from which the practice is reporting mortality in line with these statistics.

***Identifying deaths***

A flow diagram describing identification of deaths is provided as eFigure 3. We used information about deaths provided by IMRD, which takes into account information about the person’s registration status at the practice, and codes or text entries providing information about a death. We also performed an additional search for codes indicating death (Supplemental file^[[1]](#footnote-1)^), which identified additional deaths omitted by IMRD because the patient’s registration status did not indicate that they had been transferred out of the practice due to their death. For these individuals, we used the earliest of the first instance of a Read code pertaining to the death, or the person’s transfer out date, as the date of death.

We counted records as deaths that occurred up to six months after the date that the person’s records were transferred out of the practice, to allow for administrative delays in recording. We further validated the recording of all deaths by checking for non-administrative medical or prescribing records more than six months after the supposed date of death.

As a sensitivity analysis, we used a list of codes to identify possible deaths.^[[2]](#footnote-2)^ We identified possible deaths by finding occurrences of these codes that were up to 30 days before the date that the person was transferred out of the practice (suggesting that the potentially fatal event may have resulted in death), or up to six months after the date that the person was were transferred out.

We repeated these the main analyses additionally including additional possible deaths. Demographics and information about co-occurring conditions in deceased people with diagnosed ADHD plus their respective comparison groups can be found in eTables 1 - 4. Adding additional possible deaths had a negligible impact on mortality rates and life expectancy (see eTables 5 & 6).

**References**

1 Ohneberg K, Beyersmann J, Schumacher M. Exposure density sampling: Dynamic matching with respect to a time‐dependent exposure. *Stat Med* 2019; **38**: 4390–403.

2 Horsfall L, Walters K, Petersen I. Identifying periods of acceptable computer usage in primary care research databases. *Pharmacoepidemiol Drug Saf* 2013; **22**. DOI:10.1002/pds.3368.

3 Maguire A, Blak BT, Thompson M. The importance of defining periods of complete mortality reporting for research using automated data from primary care. *Pharmacoepidemiol Drug Saf* 2009; **18**. DOI:10.1002/pds.1688.

1. Death-related Read codes were identified by searching for codes containing the words “died”, “death”, “dead”, “decease”, “fatal”, “decedent”, “inquest”, “mortem”, “coroner”; and then excluding codes that pertained to someone else having died. [↑](#footnote-ref-1)
2. Possible death Read codes were identified by searching for codes containing the words “suicide”, “drowning”, “drowned”, “suffocat”, “hanging”, “hanged”, “murder”, “arrest”, “overdose”; and then excluding codes that were ambiguous or pertained to someone else. Codes indicating suicide and self-injury from an existing code-list were also included, with codes pertaining to specific forms of self-mutilation excluded. [↑](#footnote-ref-2)
