# Supplementary material for: Life expectancy and years of life lost for adults with diagnosed ADHD in the UK: matched cohort study
Source: Br J Psychiatry. 2025 Jan 23;226(5):261–8. doi: 10.1192/bjp.2024.199 (PMC7617439; doi:10.1192/bjp.2024.199)
Supplement: O'Nions et al. supplementary material 3 — O'Nions et al. supplementary material [file S0007125024001995sup003.docx]

**Supplementary Tables for “Life expectancy and years of life lost for adults with diagnosed ADHD in the UK: a matched cohort study”**

Contents

[eTable 1: Demographics of deceased males with ADHD and deceased comparison males (definite deaths) 2](#_Toc143006362)

[eTable 2: Demographics of deceased females with ADHD and deceased comparison females (definite deaths) 3](#_Toc143006363)

[eTable 3: Demographics of deceased males with ADHD and deceased comparison males (definite and possible deaths) 4](#_Toc143006364)

[eTable 4: Demographics of deceased females with ADHD and deceased comparison females (definite and possible deaths) 5](#_Toc143006365)

[eTable 5: Crude mortality rate per 100,000 people by sex and age-band people with ADHD: definite and possible deaths 6](#_Toc143006366)

[eTable 6: Mortality ratios and life expectancies by age-band: definite only and definite + possible deaths. 95% CIs are provided in parentheses. 7](#_Toc143006367)

## eTable 1: Demographics of deceased males with ADHD and deceased comparison males (definite deaths)

|  | **Deceased males with diagnosed ADHD**  **n(%)** | **Deceased males from the comparison group**  **n(%)** |
| --- | --- | --- |
| N individuals | 193 of 23377 (0.83) | 1219 of 233770 (0.52) |
| N practices | 157 | 418 |
| ***Age at cohort entry*** |  |  |
| Median age at entry (IQR) | 33.50 (19.38 - 58.42) | 36.50 (18.27 - 69.01) |
| 18 - 24 years | 71 (36.79) | 488 (40.03) |
| 25 - 34 years | 28 (14.51) | 111 (9.11) |
| 35 - 44 years | 26 (13.47) | 80 (6.56) |
| 45 - 54 years | 13 (6.74) | 93 (7.63) |
| 55 - 64 years | 15 (7.77) | 91 (7.47) |
| 65+ years | 40 (20.73) | 356 (29.20) |
| Median age at death (IQR) | 38.93 (24.74 - 60.94) | 42.12 (23.95 - 74.60) |
| ***Socioeconomic status*** |  |  |
| n Townsend score 1 (%) | 16 (8.29) | 175 (14.36) |
| n Townsend score 2 (%) | 32 (16.58) | 159 (13.04) |
| n Townsend score 3 (%) | 30 (15.54) | 228 (18.70) |
| n Townsend score 4 (%) | 34 (17.62) | 248 (20.34) |
| n Townsend score 5 (%) | 39 (20.21) | 208 (17.06) |
| n Townsend score missing (%) | 42 (21.76) | 201 (16.49) |
| ***Long-term conditions*** |  |  |
| n diabetes ever (%) | 30 (15.54) | 215 (17.64) |
| n hypercholesterolemia ever (%) | 17 (8.81) | 137 (11.24) |
| n hypertension ever (%) | 39 (20.21) | 305 (25.02) |
| n ischemic heart disease ever (%) | 20 (10.36) | 175 (14.36) |
| n chronic respiratory disease ever (%) | 38 (19.69) | 205 (16.82) |
| n epilepsy ever (%) | 24 (12.44) | 59 (4.84) |
| ***Mental health/ neurodevelopmental conditions*** | |  |
| n anxiety ever (%) | 41 (21.24) | 180 (14.77) |
| n depression ever (%) | 70 (36.27) | 265 (21.74) |
| n SMI ever (%) | 31 (16.06) | 47 (3.86) |
| n self-harm/suicide ever (%) | 50 (25.91) | 132 (10.83) |
| n autism ever (%) | 7 (3.63) | 8 (0.66) |
| n intellectual disability ever (%) | 24 (12.44) | 29 (2.38) |
| n personality disorder ever (%) | 10 (5.18) | 15 (1.23) |
| ***Lifestyle variables*** |  |  |
| n smoker/ex-smoker ever (%) | 121 (62.69) | 698 (57.26) |
| n harmful alcohol use ever (%) | 42 (21.76) | 217 (17.80) |

Note: ** indicates information redacted. SMI: Severe mental illness; “Ever” includes records after the date of death.

## eTable 2: Demographics of deceased females with ADHD and deceased comparison females (definite deaths)

|  | | **Deceased females with diagnosed ADHD**  **n(%)** | **Deceased females from the comparison group**  **n(%)** |
| --- | --- | --- | --- |
| N individuals | | 148 of 6662 (2.22) | 902 of 66620 (1.35) |
| N practices | | 105 | 236 |
| ***Age at cohort entry*** | |  |  |
| Median age at entry (IQR) | | 73.50 (57.59 - 82.67) | 77.50 (63.37 - 85.03) |
| 18 - 24 years | | 8 (5.41) | 41 (4.55) |
| 25 - 34 years | | 9 (6.08) | 28 (3.10) |
| 35 - 44 years | | ** (<4) | 31 (3.44) |
| 45 - 54 years | | 14 (9.46) | 48 (5.32) |
| 55 - 64 years | | 22 (14.86) | 95 (10.53) |
| 65+ years | | 91 (61.49) | 659 (73.06) |
| Median age at death (IQR) | | 77.22 (62.68 - 85.55) | 82.97 (70.52 - 89.69) |
| ***Socioeconomic status*** | |  |  |
| n Townsend score 1 (%) | | 23 (15.54) | 156 (17.29) |
| n Townsend score 2 (%) | | 28 (18.92) | 175 (19.40) |
| n Townsend score 3 (%) | | 17 (11.49) | 164 (18.18) |
| n Townsend score 4 (%) | | 23 (15.54) | 158 (17.52) |
| n Townsend score 5 (%) | | 31 (20.95) | 129 (14.30) |
| n Townsend score missing (%) | | 26 (17.57) | 120 (13.30) |
| ***Long-term conditions*** | |  |  |
| n diabetes ever (%) | | 28 (18.92) | 199 (22.06) |
| n hypercholesterolemia ever (%) | | 28 (18.92) | 173 (19.18) |
| n hypertension ever (%) | | 62 (41.89) | 444 (49.22) |
| n ischemic heart disease ever (%) | | 41 (27.70) | 199 (22.06) |
| n chronic respiratory disease ever (%) | | 49 (33.11) | 254 (28.16) |
| n epilepsy ever (%) | | 11 (7.43) | 33 (3.66) |
| ***Mental health/ neurodevelopmental conditions*** | | |  |
| n anxiety ever (%) | 51 (34.46) | | 190 (21.06) |
| n depression ever (%) | 69 (46.62) | | 281 (31.15) |
| n SMI ever (%) | 30 (20.27) | | 42 (4.66) |
| n self-harm/suicide ever (%) | 23 (15.54) | | 54 (5.99) |
| n autism ever (%) | ** (<4) | | 0 (0.00) |
| n intellectual disability ever (%) | 7 (4.73) | | 14 (1.55) |
| n personality disorder ever (%) | 13 (8.78) | | 12 (1.33) |
| ***Lifestyle variables*** |  | |  |
| n smoker/ex-smoker ever (%) | 72 (48.65) | | 441 (48.89) |
| n harmful alcohol use ever (%) | 23 (15.54) | | 83 (9.20) |

Note: ** indicates information redacted. SMI: Severe mental illness; “Ever” includes records after the date of death.

## eTable 3: Demographics of deceased males with ADHD and deceased comparison males (definite and possible deaths)

|  | **Deceased males with diagnosed ADHD**  **n(%)** | **Deceased males from the comparison group**  **n(%)** |
| --- | --- | --- |
| N individuals | 222 of 23377 (0.95) | 1257 of 233770 (0.54) |
| N practices | 171 | 430 |
| ***Age at cohort entry*** |  |  |
| Median age at entry (IQR) | 28.90 (18.22 - 54.84) | 34.00 (18.11 - 68.23) |
| 18 - 24 years | 93 (41.89) | 517 (41.13) |
| 25 - 34 years | 32 (14.41) | 118 (9.39) |
| 35 - 44 years | 27 (12.16) | 80 (6.36) |
| 45 - 54 years | 15 (6.76) | 94 (7.48) |
| 55 - 64 years | 15 (6.76) | 92 (7.32) |
| 65+ years | 40 (18.02) | 356 (28.32) |
| Median age at death (IQR) | 32.67 (23.49 - 57.13) | 39.44 (23.67 - 73.86) |
| ***Socioeconomic status*** |  |  |
| n Townsend score 1 (%) | 17 (7.66) | 181 (14.40) |
| n Townsend score 2 (%) | 34 (15.32) | 162 (12.89) |
| n Townsend score 3 (%) | 41 (18.47) | 233 (18.54) |
| n Townsend score 4 (%) | 42 (18.92) | 254 (20.21) |
| n Townsend score 5 (%) | 45 (20.27) | 220 (17.50) |
| n Townsend score missing (%) | 43 (19.37) | 207 (16.47) |
| ***Long-term conditions*** |  |  |
| n diabetes ever (%) | 31 (13.96) | 219 (17.42) |
| n hypercholesterolemia ever (%) | 18 (8.11) | 137 (10.90) |
| n hypertension ever (%) | 40 (18.02) | 306 (24.34) |
| n ischemic heart disease ever (%) | 21 (9.46) | 176 (14.00) |
| n chronic respiratory disease ever (%) | 40 (18.02) | 205 (16.31) |
| n epilepsy ever (%) | 26 (11.71) | 61 (4.85) |
| ***Mental health/ neurodevelopmental conditions*** | | |
| n anxiety ever (%) | 50 (22.52) | 185 (14.72) |
| n depression ever (%) | 76 (34.23) | 277 (22.04) |
| n SMI ever (%) | 34 (15.32) | 50 (3.98) |
| n self-harm/suicide ever (%) | 77 (34.68) | 167 (13.29) |
| n autism ever (%) | 8 (3.60) | 9 (0.72) |
| n intellectual disability ever (%) | 28 (12.61) | 30 (2.39) |
| n personality disorder ever (%) | 12 (5.41) | 18 (1.43) |
| ***Lifestyle variables*** |  |  |
| n smoker/ex-smoker ever (%) | 144 (64.86) | 724 (57.60) |
| n harmful alcohol use ever (%) | 50 (22.52) | 230 (18.30) |

Note: ** indicates information redacted. SMI: Severe mental illness; “Ever” includes records after the date of death.

## eTable 4: Demographics of deceased females with ADHD and deceased comparison females (definite and possible deaths)

|  | | **Deceased females with diagnosed ADHD**  **n(%)** | **Deceased females from the comparison group**  **n(%)** |
| --- | --- | --- | --- |
| N individuals | | 156 of 6662 (2.34) | 920 of 66620 (1.38) |
| N practices | | 109 | 243 |
| ***Age at cohort entry*** | |  |  |
| Median age at entry (IQR) | | 71.50 (54.50 - 81.98) | 77.39 (62.52 - 84.84) |
| 18 - 24 years | | 11 (7.05) | 54 (5.87) |
| 25 - 34 years | | 12 (7.69) | 32 (3.48) |
| 35 - 44 years | | 4 (2.56) | 31 (3.37) |
| 45 - 54 years | | 14 (8.97) | 48 (5.22) |
| 55 - 64 years | | 23 (14.74) | 95 (10.33) |
| 65+ years | | 92 (58.97) | 660 (71.74) |
| Median age at death (IQR) | | 76.11 (60.96 - 85.07) | 82.65 (68.94 - 89.62) |
| ***Socioeconomic status*** | |  |  |
| n Townsend score 1 (%) | | 23 (14.74) | 159 (17.28) |
| n Townsend score 2 (%) | | 29 (18.59) | 176 (19.13) |
| n Townsend score 3 (%) | | 20 (12.82) | 167 (18.15) |
| n Townsend score 4 (%) | | 24 (15.38) | 164 (17.83) |
| n Townsend score 5 (%) | | 33 (21.15) | 133 (14.46) |
| n Townsend score missing (%) | | 27 (17.31) | 121 (13.15) |
| ***Long-term conditions*** | |  |  |
| n diabetes ever (%) | | 29 (18.59) | 200 (21.74) |
| n hypercholesterolemia ever (%) | | 28 (17.95) | 173 (18.80) |
| n hypertension ever (%) | | 63 (40.38) | 446 (48.48) |
| n ischemic heart disease ever (%) | | 41 (26.28) | 200 (21.74) |
| n chronic respiratory disease ever (%) | | 50 (32.05) | 256 (27.83) |
| n epilepsy ever (%) | | 13 (8.33) | 34 (3.70) |
| ***Mental health/ neurodevelopmental conditions*** | | |  |
| n anxiety ever (%) | 54 (34.62) | | 196 (21.30) |
| n depression ever (%) | 75 (48.08) | | 292 (31.74) |
| n SMI ever (%) | 32 (20.51) | | 42 (4.57) |
| n self-harm/suicide ever (%) | 30 (19.23) | | 68 (7.39) |
| n autism ever (%) | 2 (1.28) | | 0 (0.00) |
| n intellectual disability ever (%) | 9 (5.77) | | 14 (1.52) |
| n personality disorder ever (%) | 14 (8.97) | | 12 (1.30) |
| ***Lifestyle variables*** |  | |  |
| n smoker/ex-smoker ever (%) | 77 (49.36) | | 455 (49.46) |
| n harmful alcohol use ever (%) | 25 (16.03) | | 88 (9.57) |

Note: ** indicates information redacted. SMI: Severe mental illness; “Ever” includes records after the date of death.

## eTable 5: Crude mortality rate per 100,000 people by sex and age-band people with ADHD: definite and possible deaths

| Age-band | **Mortality rate per 100,000** | |
| --- | --- | --- |
|  | Males with diagnosed ADHD | Comparison males |
| 18 - 24 | 126.57 (98.48 to 160.18) | 61.28 (55.11 to 67.95) |
| 25 - 34 | 215.81 (160.18 to 284.52) | 82.55 (72.26 to 93.88) |
| 35 – 44 | 405.02 (250.71 to 619.12) | 131.76 (103.81 to 164.92) |
| 45 - 54 | 762.00 (471.69 to 1164.81) | 289.42 (233.32 to 354.95) |
| 55 - 64 | 1545.42 (915.91 to 2442.43) | 673.74 (540.36 to 830.07) |
| 65+ | 4793.19 (3468.86 to 6456.40) | 3814.94 (3454.13 to 4203.20) |
|  | Females with diagnosed ADHD | Comparison females |
| 18 - 24 | 79.50 (34.32 to 156.64) | 31.26 (21.78 to 43.48) |
| 25 - 34 | 230.05 (118.87 to 401.86) | 59.24 (41.49 to 82.01) |
| 35 – 44 | 184.58 (59.93 to 430.74) | 99.91 (66.91 to 143.49) |
| 45 - 54 | 370.33 (148.89 to 763.03) | 136.59 (90.76 to 197.40) |
| 55 - 64 | 1477.43 (860.66 to 2365.51) | 544.21 (422.60 to 689.91) |
| 65+ | 6964.85 (5707.86 to 8416.31) | 3980.55 (3695.84 to 4281.36) |

## eTable 6: Mortality ratios and life expectancies by age-band: definite only and definite + possible deaths. 95% CIs are provided in parentheses.

|  | **Male** | **Female** |
| --- | --- | --- |
| **(A) Definite deaths** |  |  |
| Diagnosed ADHD | 73.26 years (71.06, 75.41) | 75.15 years (72.99, 77.11) |
| Comparison group | 80.03 years (79.34, 80.74) | 83.79 years (83.12, 84.44) |
| Difference | 6.78 years (4.50, 9.11) | 8.64 years (6.55, 10.91) |
| Mortality ratio | 1.89 (1.62, 2.19) | 2.13 (1.79, 2.53) |
| **(B) Definite + possible deaths** |  |  |
| Diagnosed ADHD | 72.80 years (70.71, 74.94) | 74.60 years (72.35, 76.56) |
| Comparison group | 79.97 years (79.27, 80.67) | 83.65 years (82.98, 84.30) |
| Difference | 7.16 years (4.87, 9.40) | 9.06 years (6.96, 11.44) |
| Mortality ratio | 2.10 (1.82, 2.42) | 2.19 (1.85, 2.60) |
